# Longterm course of neuropsychological symptoms and ME/CFS after SARS-CoV-2-infection: a prospective registry study

**DOI:** 10.1007/s00406-023-01661-3

**Published:** 2023-08-16

**Authors:** P. A. Reuken, B. Besteher, K. Finke, A. Fischer, A. Holl, K. Katzer, K. Lehmann-Pohl, C. Lemhöfer, M. Nowka, C. Puta, M. Walter, C. Weißenborn, A. Stallmach

**Affiliations:** 1 Department of Internal Medicine IV (Gastroenterology, Hepatology and Infectious Diseases), Jena University Hospital/Friedrich-Schiller-University Jena, Am Klinikum 1, 07743 Jena, Germany; 2 Department of Psychiatry and Psychotherapy, Jena University Hospital/Friedrich-Schiller-University Jena, Jena, Germany; 3 Department of Neurology, Jena University Hospital/Friedrich-Schiller-University Jena, Jena, Germany; 4 Center for Sepsis Control and Care (CSCC), Jena University Hospital/Friedrich-Schiller-University Jena, Jena, Germany; 5 Institute of Physical and Rehabilitation Medicine, Jena University Hospital/Friedrich-Schiller-University Jena, Jena, Germany; 6 Department of Sports Medicine and Health Promotion, Friedrich-Schiller-University Jena, Jena, Germany; 7 Center for Interdisciplinary Prevention of Diseases Related to Professional Activities, Jena, Germany

**Keywords:** Post-COVID, Long-COVID, SARS-CoV2, Fatigue, ME/CFS

## Abstract

A significant proportion of patients after SARS-CoV-2 infection suffer from long-lasting symptoms. Although many different symptoms are described, the majority of patients complains about neuropsychological symptoms. Additionally, a subgroup of patients fulfills diagnostic criteria for ME/CFS. We analyzed a registry of all patients presenting in the out-patients clinic at a German university center. For patients with more than one visit, changes in reported symptoms from first to second visit were analyzed. A total of 1022 patients were included in the study, 411 of them had more than one visit. 95.5% of the patients reported a polysymptomatic disease. At the first visit 31.3% of the patients fulfilled ME/CFS criteria after a median time of 255 days post infection and and at the second visit after a median of 402 days, 19.4% still suffered from ME/CFS. Self-reported fatigue (83.7–72.7%) and concentration impairment (66.2–57.9%) decreased from first to second visit contrasting non-significant changes in the structured screening. A significant proportion of SARS-CoV-2 survivors presenting with ongoing symptoms present with ME/CFS. Although the proportion of subjective reported symptoms and their severity reduce over time, a significant proportion of patients suffer from long-lasting symptoms necessitating new therapeutic concepts.

## Introduction

Shortly after occurrence of the first cases of the new form of severe acute respiratory syndrome (SARS) caused by a coronavirus (SARS-CoV-2) in December 2019 it became obvious that a relevant proportion of patients suffers from long-term sequelae, which are named post-COVID when lasting 12 weeks or longer [1]. A recent survey of our group revealed at least 20.7% of infected patients who suffer from any symptom in a population-based approach [2], which is in line with current studies from the United Kingdom reporting 13.3% of patients suffering from long-lasting symptoms [3]. Although the frequency of patients developing long term sequelae is lower in vaccinated individuals [4] and in patients infected with less pathogenic variants including omicron [5], the burden for health care systems is still high due to the increasing number of acute cases. Furthermore, DeVries et al. very recently described increased 12-month mortality rates in patients with post-COVID syndrome compared to patients with an acute SARS-CoV-2-infection without developing a post-COVID syndrome [6].

Until today, up to 200 different symptoms of different intensity, frequency and duration have been described [7], e. g., respiratory, psychiatric, cognitive, cardiovascular, gastrointestinal or inflammatory symptoms. Neuropsychological symptoms are the most frequent problems in these patients [8, 9], including fatigue, sleep disturbance, cognitive dysfunctions and depression [10].

A substantial problem in patients with post-COVID is the transition to ME/CFS. ME/CFS is a multi-systemic disease with several neuropsychological symptoms occurring in 0.17 to 0.89% of the population with a predominance in women [11]. Although the pathophysiology resulting in ME/CFS is not fully understood, viral infections are regarded an important trigger [12]. While Oliveira et al. reported “nearly all” patients with long-term sequelae of SARS-CoV-2 would meet diagnostic criteria for ME/CFS, other studies report that only 13–25% of the patients meet the ME/CFS criteria [13–15].

Therefore, the aim of our study was to analyze potential change in neuropsychological symptoms over time with special reference to ME/CFS in post-COVID patients during long-term follow-up in a single-center cohort of post-COVID patients.

## Methods

All patients presenting before October 31st 2022 to the post-COVID outpatient clinic of Jena University Hospital, which was established in August 2020, were prospectively included into the analysis. At the first visit, detailed information on demographics, course and treatment of the acute SARS-CoV-2 infection and preexisting health condition was performed. The infection severity was classified with a modified 10-point scale from the WHO [16]. Furthermore, at every visit, all patients received a structured assessment consisting of body examination, self-reported symptoms evaluation and structured psychiatric and cognitive screening. The following post-covid associated self-reported symptoms were assessed: Fatique was assessed using Fatigue Assessment Scale, FAS and the Brief Fatigue Inventory, BFI [17, 18]. In the structured screening, fatigue was defined as ≥ 22 points in the FAS or a mean level of ≥ 1 in the BFI. Severity of fatigue was defined via the mean BFI (< 1: no Fatigue, 1 to < 4: mild Fatigue, 4 to < 7: moderate Fatigue, 7–10: severe Fatigue) [17, 19, 20]. In case a patient answered more than one of the fatigue questionnaires, the worst result was used. The Depression module of the Patient Health Questionnaire, PHQ-9 was used to assess Depression [21]. According to PHQ9, patients depressive symptoms were classified as follows: minimal (1–4), mild (5–9), moderate (10–14), moderately severe (15–19), or severe depressive symptoms (20–27). [21] Results of ≥ 5 points in the PHQ-9 were regarded as clinically relevant. All questionnaires and tests were used in the German version and interpretation was performed according to the manuals. Cognitive dysfunction was assessed using the “Montreal Cognitive Assessment” (MoCA) screening [22]. Cognitive dysfunction was defined as a MoCA test results < 26 points [22]. Post exertional malaise as cardinal sign of ME/CFS was assessed according to Cotler et al. [23]. Diagnosis of ME/CFS was based on Canadian Consensus Criteria (CCC) and exclusion of other chronic fatigue related diseases [22].

The study was approved by the institutional ethics committee of Friedrich-Schiller-University Jena (2020-1978-Daten).

### Statistical analyses

Statistical analyses were performed using SPSS v28 (IBM Inc, Armonk, NY) and PRISM 9 (Graphpad Inc, LaJolla, CA). We summarized the patient characteristics as absolute and relative frequencies for categorical variables, and as medians and first and third quartiles (Q1, Q3) for numerical variables, unless stated otherwise. For explorative comparisons between two patient groups, Fisher’s exact test for categorical variables and the Mann–Whitney U test for numerical variables were used. For comparison between the first and the second visit McNemar test was used for categorial variables and Wilcoxon test for numerical variables. In time to event comparisons, the log rank test and corresponding Kaplan–Meier curve were used.

## Results

### Overall

Between August 1st 2020 and October 31st 2022, 1.022 patients visited our post-COVID out-patient clinic. 684 (66.9%) of the patients were female with a median age of 51 years (41–59). The median time between infection and the first presentation to the post-COVID out-patient clinic was 255 days (84–728 days) after diagnosis of SARS-CoV-2 infection. Symptoms lasted for more than one year in 270 patients at initial presentation. A total of 204 patients (20.0%) required hospitalization for treatment of the acute infection, including 124 patients (12.1% of all patients and 60.8% of hospitalized patients) who needed oxygen supply. In detail, according to the WHO stage, 32 patients (3.1%) were classified as stage 1, 733 patients as stage 2 (71.6%) and 53 patients (5.2%) as stage 3. Among hospitalized patients, 80 patients (7.8% of all patients and 39.2% of the hospitalized patients) did not receive oxygen supply (WHO stage 4) and 76 patients (7.4% of all patients and 37.2% of hospitalized patients) required low-flow oxygen (WHO stage 5) and 48 patients (4.7% of all patients and 23.5% of hospitalized patients) were treated on an ICU (WHO-Stage 5–9).

### First and second visit

Four-hundred eleven patients (40.2%) visited the out-patient clinic within the study period more than once. The duration between the initial presentation in the outpatient clinic and the second visit was 147 (119–202) days, resulting in a median time between infection and the second visit of 374 (288–466) days. Of these 411 patients, 271 were female (65.9%), and the median age was 52 (43–60) years.

Of the patients with more than one visit to the post-COVID out-patient clinic, 327 (79.6%) were treated as outpatients during the acute infection and 84 patients (20.4%) required hospitalization, including 77 patients (18.7% of the 411 patients) with need for oxygen supply. Detailed information on demographics and the course of the initial infection is presented in Table 1.

**Table 1:** Demographics and disease course of post-COVID patients

Characteristic	All patients (n = 1022)	Patients with more than one visit (n = 411)
Female sex (n)	684 (66.9%)	271 (65.9%)
Age (years)	51 (41; 59)	52 (43; 60)
Days since infection	255 (84; 728)	374 (288; 466)
Out-patient only (n)	818 (80.0%)	327 (79.6%)
Among them:		
WHO grade		
1 (n)	32 (3.1%)	15 (3.7%)
2 (n)	733 (71.6%)	282 (68.6%)
3 (n)	53 (5.2%)	30 (7.3%)
In-patient; n (%)	204 (20.0%)	84 (27.4%)
Among them:		
In need of oxygen support; n (%)	124 (12.1%)	47 (11.4%)
ICU stay; n (%)	48 (4.7%)	18 (4.4%)
WHO grade:		
4 (n)	80 (7.8%)	37 (9.0%)
5 (n)	76 (7.4%)	29 (7.1%)
6 (n)	18 (1.8%)	6 (1.5%)
7 (n)	13 (1.3%)	7 (1.7%)
8 (n)	6 (0.6%)	1 (0.2%)
9 (n)	11 (1.1%)	4 (1.0%)

Absolute (n) and relative frequencies (%) or median together with first and third quartile (Q1, Q3) are provided

*ICU* intensive care unit, *N* number of patients in total, *WHO* World Health Organization

### Self-reported symptoms

All patients received a structured screening of self-reported symptoms at every visit. As patients were included when making an appointment in the post-COVID outpatient clinic, every patient reported at least one symptom. The vast majority of patients reported more than one major symptom (942 patients, 92.2%). The most frequent symptoms were neuropsychological symptoms including self-reported fatigue in 809 patients (79.1%), concentration (653 patients, 93.9%) and memory impairment (555 patients, 54.3%). Signs of depression were reported by 273 patients (26.7%), persisting headache by 337 patients (33.0%), smell/taste disorder by 296 patients (28.6%) and sleeping disorder by 381 patients (37.3%). The most frequent somatic symptoms were dyspnea (542 patients, 53.0%) and muscle pain (309 patients, 30.2%).

Focusing on the first visit of the 411 patients with more than one visit, again, the vast majority reported polysymptomatic disease (394 patients, 95.9%), most frequently fatigue (344 patients, 83.7%), concentration (272 patients, 66.2%) and memory impairment (229 patients 55.7%). Sleeping disorder was reported by 193 patients (46.9%), headache by 161 patients (39.2%), smell/taste disorder by 131 patients (31.9%) and depression by 123 patients (29.9%). The most frequent somatic symptom was dyspnea reported by 253 patients (61.6%). In the first follow-up visit, still, the vast majority suffered from symptoms of post-COVID syndrome. Compared to the initial visit, the self-reported symptoms revealed a reduction in the patients suffering from self-reported fatigue (299 patients, 72.7%, p = 0.002 compared to the first visit) and sleep disorder (170 patients, 41.4%, p = 0.007) as well as an unchanged proportion in self-reported concentration impairment (238 patients, 57.9%, p = 0.411), while the other neurological symptoms remained unchanged (memory impairment 57.2%, p = 0.342; depression 31.9%, p = 0.07; headache 36.0%, p = 0.670). The rate of patients reporting dyspnea decreased to 41.6% (171 patients, p < 0.001) (Table 2).

**Table 2:** Self-reported symptoms of patients with more than one visit (n = 411)

	First visit	Second visit	p-value
Fatigue	344 (83.7%)	299 (72.7%)	0.002
Impairment in concentration	272 (66.2%)	238 (59.9%)	0.411
Impairment in memory	229 (55.7%)	235 (57.2%)	0.170
Sleeping disorder	193 (46.9%)	170 (41.4%)	0.007
Headache	161 (39.2%)	148 (36.0%)	0.670
Smell/taste disorder	131 (31.9%)	114 (27.7%)	0.541
Depression	123 (29.9%)	131 (31.9%)	0.070
Dyspnea	253 (61.6%)	171 (41.6%)	< 0.001

Absolute (n) and relative frequencies (%)are provided

### Neuro-psychiatric and cognitive screening

**Fig. 1:**
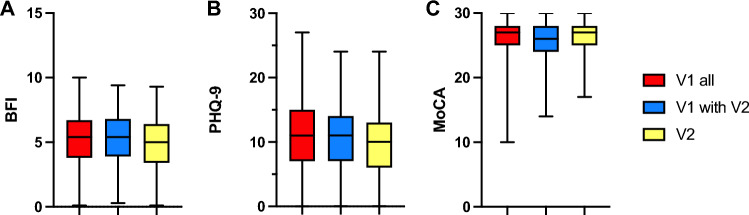
Results of the structured screening for Fatigue (BFI, Fig. 2A), sings of depression (PHQ-9, Fig. 2B) and memory impairment (MoCA, Fig. 2C) in 1.022 post-COVID patients at the first visit (V1 all), and in the subgroup of patients with more than one presentation at the outpatient clinic at the first (V1 with V2) and second (V2) visit

The structured psychiatric screening revealed pathological results defined as hints for fatigue or depression in 876 patients (85.7% of all patients). Chronic fatigue as indicated by FAS was the most frequent symptom found in 872 patients (85.3%). According to BFI, it was classified as “mild” in 191 patients (18.7% of all patients), as “moderate” in 482 patients (47.2%) and as “severe” in 199 patients (19.4%). The median BFI Score was 5.4 (0.1–10). Looking at the 411 patients with more than one presentation at the post-COVID outpatient clinic, 351 patients (85.4%) had signs of fatigue or depression at the first visit, including 347 patients (84.4%) with fatigue. Of these patients, 58 patients (14.1%) suffered from “mild fatigue”, 197 patients (47.9%) from “moderate fatigue” and 92 patients (22.4%) from “severe fatigue”. Contrasting the decline in self-reported fatigue between the first and follow-up visit, 342 patients (83.2%) still showed signs of fatigue (p = 0.222) in the structured screening. However, the severity of fatigue was significantly reduced compared to the first visit. Overall, 113 patients (27.5%) reported “mild fatigue”, 169 patients (41.1%) “moderate fatigue” and only 60 patients (14.6%) severe fatigue (p < 0.001) with a median BFI score slightly decreased to 5.0 points (Fig. 1A).

Contrasting the self-reported depression by 273 patients (26.7%), the structural screening revealed sings of depression in 843 patients (82.5%), including 290 patients (28.4%) with “mild depression, 304 patients (29.7%) with “moderate depression” and 249 patients (24.4%) with “severe depression”, with a median PHQ-9 score of 11 points. Focusing on the patients with more than one visit, as for the fatigue, 347 (84.4%) patients had signs of depression according to PHQ9. 114 patients (27.7%) had sings of “mild depression”, 141 patients (34.3%) of “moderate depression” and 92 patients (22.4%) of “severe depression” with a median PHQ9 score of 11 points. At the second visit, still 310 patients (75.4%) had pathological PHQ9 screening indicating depression symptoms. As for the fatigue screening, the severity of depression symptoms was reduced compared to the first visit (p < 0.001), including 124 patients (30.2%) with “mild “symptoms, 115 patients (28.0%) with “moderate” symptoms and 71 patients (17.3%) with “severe” symptoms (Fig. 1B).

The cognitive screening with the MoCA score indicated cognitive dysfunctions in 267 patients (26.1%) with a median score of 26.5 points, which is at the lower end of the the normal range of 26 or more points. Only focusing on the patients with more than one visit, 133 of the 411 patients (27.5%) had pathological results in the MoCA score (median 26 points), while in the second visits, only 89 patients (21.7%) had pathological results in the MoCA and the median slightly improved to 27 points (Fig. 1C).

### Myalgic encephalomyelitis/chronic fatigue syndrome

**Fig. 2:**
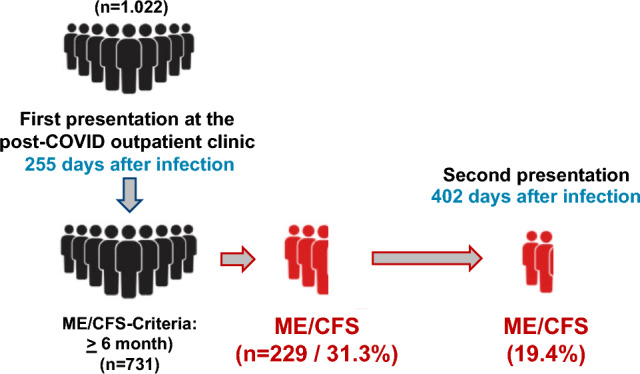
Visualization of the proportion of patients fulfilling the Canadian Criteria for ME/CFS at the first and the second visit in the post-COVID outpatient clinic

Overall, 731 out of the 1022 patients presented at least 180 days or more after the acute infection. 229 (31.3%) of the 731 patients fulfilled the Canadian Consensus Criteria (CCC) for ME/CFS [22] (Fig. 2). During follow-up, additional 75 patients presenting before day 180 at the first visit fulfilled the diagnostic criteria for ME/CFS resulting in a total of 304 patients with ME/CFS in the total cohort of 1022 patients (29.7%). As the definition of ME/CFS includes several mandatory conditions, a relevant proportion of patients suffered from several ME/CFS-symptoms but failed the ME/CFS criteria, most frequently, because sleep disturbance was not reported by the patients.

Four hundred and eleven patients presented more than once at the post-COVID outpatient clinic. Of these, 120 patients (29.2%) were diagnosed with ME/CFS at the first visit. At the second visit at a median of 402 days after the infection, ME/CFS was still present in 62 of these patients (51.6% of the initial ME/CFS patients) and improved in 58 patients (48.4% of the initial ME/CFS patients). (Fig. 2) The main improvements were a reduction of pain in 37 patients (30.8% of the initial ME/CFS patients). A reduction in cognitive dysfunction was observed in 23 patients (19.2% of the initial ME/CFS patients, including 4 patients with reduction of both, pain and cognitive dysfunction). Sleep disturbance improved in 32 patients (26.7% of the initial ME/CFS patients) and 15 patients reported a reduction of fatigue (12.5% of the initial ME/CFS patients, including 5 patients with reduced pain, 6 with improved neurological symptoms and 2 with improvement of both, pain and neurological symptoms).

## Discussion

Although the proportion of patients suffering from post-COVID symptoms and severity of individual symptoms reduce over time, patients suffer from long-lasting symptoms necessitating new therapeutic concepts. In the current study, ME/CSF was present in 31.3% of post-COVID patients at the first visit 255 days after the acute infection and in 15.1% at the second visit after a median of 402 days after the acute infection. Although the proportion of patients with ME/CFS and self-reported burden of disease decreased, the structured screening still identified a high frequency of patients suffering from neuropsychological sequelae in long-term follow up.

Until today, the pathophysiology resulting in long term sequelae after a SARS-CoV-2 infection is still unclear [24]. The main hypotheses include viral persistence, development of auto-immunity, including g-protein coupled autoantibodies [25], metabolic changes and vascular or endothelial damage [24]. Interestingly, there are several similarities between post-COVID sequelae and ME/CFS. Several studies suggest that neuropsychological sequelae after acute SARS-CoV-2 infection have similarities with the chronic fatigue syndrome (ME/CFS) [13, 14, 26]. In a small cohort study 45% of the patients with post-COVID fulfilled the diagnostic criteria for ME/CFS and additionally, similarities in the pathophysiology are discussed [15].

The results of our study are in line with results reported by Oliveira et al. who reported a significant improvement in a cohort of post-COVID patients with ME/CFS, so that these patients did not meet the ME/CFS criteria any longer [13]. In detail, the post-COVID patients improved in reported fatigue (70.6 vs. 56%) and memory impairment (53.8 vs. 38.1%), while patients with diagnosed ME/CFS outside a post-COVID syndrome did not improve within one year. In our cohort, the frequency of self-reported fatigue decreased between the first two visits as well, contrasting that, structured screening did not show a lower frequency of fatigue, but a decrease in the severity of fatigue, which may illustrate that the relevant burden of disease reported by patients does not necessarily agree with the cutoff values of structured tests. Despite the reduction in patients that meet the ME/CFS criteria in our cohort, most patients still suffered from relevant symptoms, underlining the need for sufficient long-term concepts to avoid long-lasting sequelae.

There is a high demand for new therapeutic concepts, as post-COVID conditions lead to a relevant reduction in quality of life in the affected patients and to high social and economic burden of disease. Recent cohort studies report high frequencies of patients suffering from fatigue up to one year after the acute infection [2, 27, 28]. A rehabilitation program could improve multiple symptoms in a cohort of post-COVID patients, but not their fatigue, and even after discharge from rehabilitation the majority of these patients was unable to work [29]. Current therapeutic concepts mainly consist of non-pharmacological interventions, including physical therapy, rehabilitation, neuro-cognitive training and psychological interventions [30]. In post-COVID patients suffering from ME/CFS, the use of pacing strategies, that are already well-established in the treatment of ME/CFS [31], is of central importance. Physical and cognitive pacing strategies in rehabilitation approaches balance rest and physical and mental activities in daily, to manage symptoms such as fatigue, avoid post exertional symptom exacerbation (PESE), post exertional malaise (PEM) and exercise intolerance PEM in post-COVID patients [9]. Pacing-based rehabilitation programs can help people with long COVID reduce their symptoms and maintain their physical and cognitive activity levels [32, 33]. It was described as effective in reducing symptom burden in a non-controlled case series of 31 post-COVID patients [32]. It is important to note that physical exercise can be harmful for patients with long-term post-COVID suffering from ME/CFS and particularly from post-exercise discomfort. A study in people with long COVID documented that physical activity led to a further worsening in 75% of patients, and to improvement only in 1% of patients [36]. Thus, the applicability of physical treatment in post-COVID patients is limited to cases without severe PEMs and should only be applied in combination with pacing strategies [34, 35]. It should be considered that one study of people with long COVID noted that physical activity worsened the condition of 75% of patients, and less than 1% saw improvement [36].

Several case reports are available for medical treatments based on the proposed pathophysiology e.g., oral antivirals [37] or neutralization of β-adrenergic autoantibodies [38]. Of note, the use of immunoadsorption to remove these antibodies is controversially discussed and was not successful in a recent case series [39]. However, randomized trials are missing and urgently needed. A recent review found 59 clinical trials investigating patients with post-COVID [40], including pharmacological, interventional, rehabilitative or dietary components and focusing on different symptoms of the post-COVID syndrome. One main limitation in providing care for all post-COVID patients is the availability of experienced medical personal. The use of telemedical concepts and guidance to self-training may provide a promising option to overcome this constraint [41] as these are already successfully used in other indications [42, 43].

Cognitive dysfunction was assessed using the MoCA in our cohort and revealed pathological results in 26.1% of the patients at the first visit. When interpreting these results there are some limitations that need to be taken into account. Most important the MoCA was developed as a screening in the elderly that would trigger more comprehensive neuropsychological assessment. Thus, more subtle deficits in younger post-COVID syndrome patients below the age of 65 might remain undetected. However, it is a simple and in outpatient settings widely used assessment tool, therefore we decided to include it in the analysis. Due to these limitations, a more detailed and validated assay may be useful, when patients report cognitive problems after a SARS-CoV-2 infection.

As another important limitation, we included only patients presenting to our outpatient clinic, where they presented on a symptom-driven manner, therefore, we cannot calculate prevalence of the disease. All patients received an appointment for the second visit, the difference between the whole cohort and the patients with more than one visit are explained by upcoming appointments. Overall, 38 patients did not show to the second appointment without providing a reason for non-showing or making a new appointment, therefore we cannot exclude a complete cure from post-COVID in these patients. Second, as this was not a controlled intervention trail, the therapy provided between the first and the second appointment was heterogenous, including in- and outpatient rehabilitation, physical therapy, occupational therapy, cognitive training and psychotherapy, based on the decision of the treating physician and is furthermore subject to patients’ compliance.

In conclusion, our study demonstrates the long-lasting burden, especially of neuropsychological symptoms of post-COVID. Prospective studies and specialized interventions are urgently needed to optimize care for these patients.

## Data Availability

Data underlying this manuscript is available from the corresponding author on reasonable request.
